# Contribution and Effects of PM_2.5_-Bound Lead to the Cardiovascular Risk of Workers in a Non-Ferrous Metal Smelting Area Considering Chemical Speciation and Bioavailability

**DOI:** 10.1021/acs.est.2c07476

**Published:** 2023-01-23

**Authors:** Xiaochong He, Qiting Zhao, Xuyang Chai, Yuanyuan Song, Xuelan Li, Xingwen Lu, Shoupeng Li, Xin Chen, Yong Yuan, Zongwei Cai, Zenghua Qi

**Affiliations:** † Guangdong-Hong Kong-Macao Joint Laboratory for Contaminants Exposure and Health, School of Environmental Science and Engineering, Institute of Environmental Health and Pollution Control, Guangdong University of Technology, Guangzhou510006, China; ‡ State Key Laboratory of Environmental and Biological Analysis, Department of Chemistry, Hong Kong Baptist University, Hong Kong00000, China; § The Center for Reproductive Medicine, Shunde Hospital, Southern Medical University (The First People’s Hospital of Shunde), 528300Foshan, Guangdong, China; ∥ Analysis and Test Center, Guangdong University of Technology, Guangzhou510006, China

**Keywords:** PM_2.5_-Pb, pollution characteristics, relative bioavailability, cardiovascular damage, health risk assessment

## Abstract

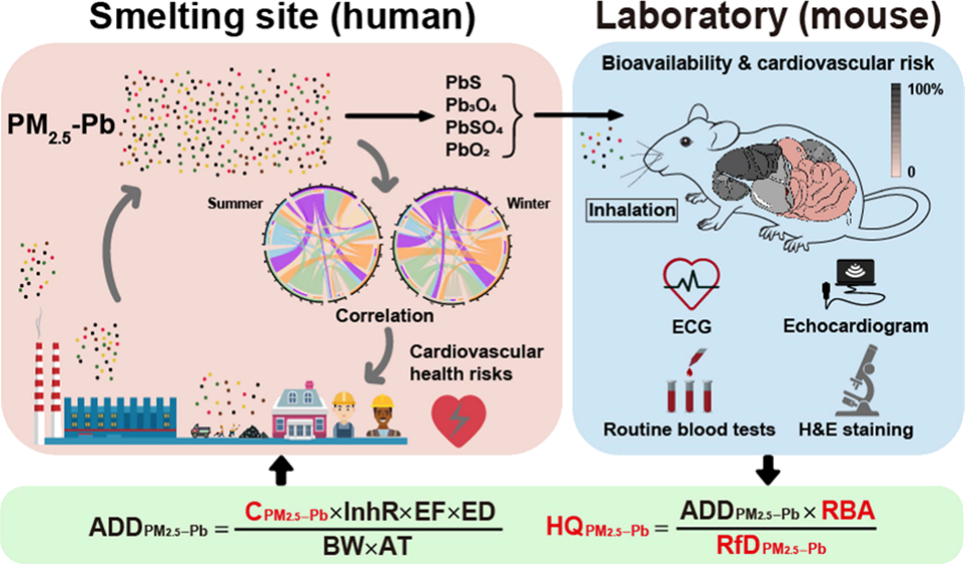

Lead is known to have toxic effects on the cardiovascular system. Owing to its high concentration, transmission range, and absorption efficiency in organisms, inhalation of fine particulate matter (PM_2.5_)-bound lead (PM_2.5_-Pb) may cause significant cardiovascular damage. However, the contribution and adverse effects of PM_2.5_-Pb on workers and residents in non-ferrous metal smelting areas are not fully understood. In this work, the concentration and chemical speciation of PM_2.5_-Pb were analyzed to determine its pollution characteristics at a typical non-ferrous metal smelting site. A panel study conducted among factory workers revealed that PM_2.5_-Pb exposure makes an important contribution to the human absorption of Pb. Although the chemical speciation of PM_2.5_-Pb suggested poor water solubility, a high bioavailability was observed in mice (tissue average value: 50.1%, range: 31.1–71.1%) subjected to inhalation exposure for 8 weeks. Based on the bioavailability data, the relationship between PM_2.5_-Pb exposure and cardiovascular damage was evaluated in animal simulation experiments. Finally, a damage threshold and cardiovascular-specific risk assessment model were established for the non-ferrous metal smelting area. Our project not only accurately estimates the risk of PM_2.5_-bound heavy metals on the cardiovascular system but also offers a scientific basis for future prevention and therapy of PM_2.5_-Pb-related diseases.

## Abstract 2

This study identified the risks of PM_2.5_-Pb on the cardiovascular system by developing a comprehensive research route covering “pollution−external exposure−internal exposure−adverse health outcome” in non-ferrous metal smelting areas.

## 1 Introduction

China is the leading producer and consumer of non-ferrous metals in the world, with the annual output of 10 non-ferrous metals reaching 6.45 million tons in 2020.^1^ During the non-ferrous metal smelting process, the main metal and its accompanying elements in ore and concentrate may be released into the surrounding environment via wastewater, waste gas, and waste residue, making non-ferrous metal smelting areas the main sites of heavy metal pollution.^2^ Upgrading the smelting system and strengthening environmental protection measures will change the transport medium and major pathway of lead (Pb) exposure for workers in smelting areas. Therefore, based on current smelting processes, prevention, and control strategies, it is important to accurately identify and implement scientific management of heavy metal pollution to clarify the exposure path and health risk of heavy metals at smelting sites.

Fine particulate matter (PM_2.5_) is an effective carrier for heavy metal diffusion due to its complex composition, small particle size, large number, and large surface area.^3,4^ Owing to environmental concerns, the exposure and adverse effects of PM_2.5_-bound heavy metals (PM_2.5_-HMs) have attracted considerable attention in the past few years. One study of heavy metals in atmospheric particles in a mining and metallurgy area in Southwest China found that Cd, Cu, Pb, and Zn were enriched in submicron particles and the deposition ratio in alveoli was relatively high, thus endangering human health.^5^ Therefore, the health risks of harmful heavy metals in smelting areas on workers and surrounding residents through the transmission path of PM_2.5_ cannot be ignored.

Particulates carrying Pb in nonferrous metal smelting areas have a complex chemical form, rich content, widespread distribution, and high body absorption rate, which may increase cardiovascular damage.^6,7^ Guo et al. reported that nine PM_2.5_-HMs were significantly associated with various cardiovascular diseases (CVDs) and CVD mortality, among which the calculated cumulative excess risk value of the CVD mortality of PM_2.5_-bound Pb (PM_2.5_-Pb) was the largest.^8^ Other studies have also shown independent associations between PM_2.5_-Pb and CVD mortality.^9^ However, the specific effects and contribution of PM_2.5_-Pb exposure on cardiovascular dysfunction in non-ferrous metal smelting areas are still unclear.

Bioavailability is an important factor for the assessment of in vivo toxicity and health risk.^10^ The reactivity and bioavailability of heavy metals in the environment are related not only to the total amount but also to their chemical speciation and exposure pathways. Despite the lack of fine chemical speciation of PM_2.5_-Pb in non-ferrous metal smelting areas, recent studies have used a variety of identification methods to investigate the speciation of Pb in inhalable (<10 μm) soil particle fractions and found that water-insoluble anglesite and lead oxide are the predominant Pb species.^11,12^ In general, water-soluble heavy metals are easily absorbed and transported by organisms. However, Zhong et al. recently investigated the inhalation relative bioavailability (RBA) of seven Pb compounds with poor water solubility in simulated PM_2.5_ and found that it was relatively high (RBA > 60.0%).^11^ In addition, previous investigations have indicated that inhalation is the main exposure pathway for PM_2.5_ pollution.^13^

Based on the potential cardiovascular risk of PM_2.5_-Pb due to its concentration, speciation, and bioavailability, we hypothesized that PM_2.5_-Pb is an important environmental risk factor for the incidence of cardiovascular injury. Thus, we conducted a comprehensive panel study and used an animal exposure model based on environmentally realistic concentrations and mixture compositions to (1) clarify the pollution characteristics of PM_2.5_-Pb in a non-ferrous metal smelting area; (2) explore the bioaccumulation, RBA, and toxic impacts on the circulatory system of PM_2.5_-Pb; and (3) establish a cardiovascular-specific risk assessment model of PM_2.5_-Pb exposure.

## 2 Materials and Methods

### 2.1 Study Area Characteristics and Sample Collection

The study site contained a large lead-zinc smelter located in Gansu Province, China. The area covered about 27.5 km^2^, and the smelter had been in operation since 1978. The main processes carried out at the smelter were purification of lead-zinc ore by blasting, smoke-return sintering, and closed blast furnace smelting by the Imperial smelting process (ISP). In addition, an acid production system at the site utilized adiabatic evaporation washing, two-rotation and two-suction processes.

From 2020 to 2022, according to the functional layout and meteorological conditions of the study site (Figure S1), four sampling points in the smelting site and surrounding area were set up to collect PM_2.5_ and PM_10_ using quartz microfiber filters (Whatman, QMA, 90 mm diameter) and a medium-volume air sampler (Laoying Co. Ltd., China) over 24 h at a flow rate of 100 L·min^–1^. Eight PM_2.5_ samples were collected each month from both the smelting and office areas. Before usage, filters were placed into a muffle oven at 450 °C for 4 h to eliminate interference. After sampling, all the filters were stored in the glass dryer until further analysis. In total, 96 samples were collected for quantitative analyses. Environmental samples such as soil, dust, minerals, and slag were collected from different functional areas of the site. In addition, the dust samples from other four non-ferrous metal smelting sites located in Sichuan Province (Southwestern China), Hunan Province (Central China), Guangdong Province (Southern China), and Guizhou Province (Southwestern China) were collected to verify the applicability of our study.

### 2.2 Pollution Profile and Chemical Speciation of PM_2.5_-Pb

PM_2.5_ mass, composition, and morphology analyses were conducted according to our previous studies.^14,15^ The specific preprocessing and pre-detection methods used for the filter membranes are presented in the Supporting Information (Section S1). After sample collection, filter membranes with retained PM_2.5_ were cut and ground into 1 cm^2^ square pieces and then added to the nitric acid solution for pre-digestion. Subsequently, the pre-digestion mixture was subjected to microwave digestion (MARS6, CEM Co. Ltd., USA). After acid removal and filtering with a 0.22 μm water-based filter, it was diluted to 50 mL of 2% nitric acid solution. Finally, an appropriate amount of an internal standard mixture (^45^Sc, ^73^Ge, ^89^Y, ^115^In, ^185^Re, ^193^Ir, ^209^Bi) was added, and the solution was mixed and left overnight before detection by inductively coupled plasma-mass spectrometry (ICP-MS; model ICAP RQ, Thermo Fisher Scientific Co. Ltd., USA). The specific parameters are shown in Table S1. Details of the quality assurance and quality control are provided in the Supporting Information (Section S2).

Surface chemical characterization of PM_2.5_-Pb was examined by means of X-ray photoelectron spectroscopy (XPS, Escalab 250Xi, Thermo Fisher Scientific Co. Ltd., USA). Filter membranes with retained PM_2.5_ were cut and ground into 1 cm^2^ square pieces, which were then mounted on a sample holder with carbon tape. Photoelectrons were generated with a monochromatic Al Kα X-ray source.

The morphology of PM_2.5_-Pb was characterized by X-ray diffraction (XRD, Ultima IV, Rigaku Co. Ltd., Japan). The sample treatment method was the same as for the XPS analysis described above. Scans were recorded for the 2θ range from 10 to 80° with a step size of 2° per minute. Crystalline phase identification and content determination of PM_2.5_-Pb in the filter membranes were performed with the X’Pert HighScore Plus software (version 3.0.5) and ICDD PDF-2 database.^16^

### 2.3 Panel Study Design and Cardiovascular Risk Assessment

A panel study was designed to investigate the contribution of PM_2.5_ to Pb accumulation in smelting area workers. The panel study was conducted during two seasons: winter (from December 1, 2020 to January 20, 2021) and summer (from July 1, 2021 to August 20, 2021). The collection time and frequency of environmental and human sampling were determined by the workers’ work schedules, and samples were taken on at least 15 days for every volunteer per season. Based on the potential exposure routes of Pb for local workers, we collected inhalation exposure source samples, dermal exposure source samples, and digestion exposure source samples, i.e., atmospheric particulate matter samples (PM_2.5_ and PM_10_), skin wipe samples, and food and drinking water samples, respectively. Dermal exposure samples were collected at the end of work shifts using Ghostwipes (SC4250, Pennsylvania, USA) to swab the palm of workers’ dominant hand and their forehead from a fixed area of 3 cm × 3 cm. Different types of food were purchased from the employee’s internal restaurant to represent the workers’ daily diet. Water samples were obtained directly from the water dispensers in the workers’ retiring room and office.

In addition, each participant collected urine at least once during working hours and mixed urine samples multiple times. After sample collection, all samples were individually packaged and placed in a −20 °C freezer. Once all samples had been collected, they were transported in dry ice to the laboratory for analysis. At the same time, research questionnaires on lifestyle, behavior habits, cardiovascular symptoms, and disease history were distributed to 150 smelting site workers, and 93 occupational health examination reports were collected. The cardiovascular risks of workers were calculated using the Prediction for Atherosclerotic Cardiovascular Disease (ASCVD) Risk in China (China-PAR) model.^17^

A total of 25 workers (18 men, 7 women) who had worked at the site for more than 1 year were recruited for our study. The study was approved by the ethical committee of Shunde Hospital, Southern Medical University (KYLS20220127), and all volunteers provided written informed consent before enrollment. The detection method of Pb in food, water, urine, and skin wipe paper was the same as Section 2.2 by ICP-MS. Creatinine was measured in all urine samples, and the urinary concentration of lead (Pb–U) was normalized according to the corresponding creatinine concentration. The software package Origin (version 2022b) was used to analyze the correlation between pollutants from various sources and the Pb content in urine.

### 2.4 Animal Simulation Exposure Experiment

To simulate as closely as possible the actual exposure scenario of Pb in PM_2.5_ at the smelting site, an animal exposure experiment was conducted. A simulated exposure solution was prepared according to the chemical speciation and proportion of PM_2.5_-Pb detected in the analysis of filter membranes. Mixed Pb standard compounds were crushed into a powder using a comet ball grinding mill (QM-3SP2, Nanjing University Instrument Co. Ltd., China) to a particle diameter < 2.5 μm and then dispersed in an appropriate volume of a 0.9% sodium chloride solution. The morphology of the simulated PM_2.5_ suspension observed using a scanning electron microscope^18^ is shown in the Supporting Information (Section S3).

Eighty-four (42 male, 42 female) C57BL/6 mice were provided from the Guangdong Medical Laboratory Animal Center and raised under specific pathogen-free (SPF) conditions with an adaptation period of 2 weeks prior to the experiment. Before starting the experiment, the body weight, blood pressure, blood glucose, and heart rate of the mice were recorded under natural conditions, and urine and feces were collected to determine baseline values. Separately reared male and female mice were exposed simultaneously to the same group settings. Mice of the same sex were randomly divided into seven groups, each comprising six mice: a control group (C-group), two low dosage groups (L_1/50_-group 0.23 μg·kg^–1^·day^–1^; L_1/5_-group 2.32 μg·kg^–1^·day^–1^, corresponding, respectively, to 1/50 and 1/5 of the mean concentration of PM_2.5_-Pb collected at the non-ferrous metal smelting site across the whole year), a medium dosage group (M-group,11.6 μg·kg^–1^·day^–1^, corresponding to the mean concentration of PM_2.5_-Pb), two high dosage groups (H-group 49.8 μg·kg^–1^·day^–1^; H_2_-group 99.6 μg·kg^–1^·day^–1^, corresponding to the maximum and two times the maximum concentration of PM_2.5_-Pb), and a lead acetate exposure group (Pb(Ac)_2_-group, corresponding to the maximum concentration of PM_2.5_-Pb). The exposure concentrations for each group can be calculated from the data presented in the Supporting Information (Section S4) and are shown in Table S2. Intratracheal instillation of the mice was performed as described previously.^10^ Briefly, 20 μL of the simulated PM_2.5_-Pb suspension or 20 μL of saline was injected through the mouth into the trachea. Instillation was performed three times per week until the cardiovascular system related indexes (heart rate, heart-rate variability, blood pressure, and blood glucose) of the living model in the experimental group were significantly different from those in the control group or the concentrations of heavy metals in urine were constant. Echocardiograms of mice in the H_2_- and C-group were recorded (see Section S5 and Table S3 for details).

The animal study was approved by the Animal Ethics Committee of Shunde Hospital of Southern Medical University, and all the operations followed the Guide for the Care and Use of Laboratory Animals.

### 2.5 Key Characteristics of Cardiovascular Damage

During the exposure experiment, the heart rate and blood pressure of mice were measured, and their urine was collected using metabolic cages weekly. Tail vein blood of mice was collected every 2 weeks to measure blood glucose. At the end of exposure, the heart function of mice was examined by echocardiography. The procedures used for the experiments are described in our previous work.^15^

Plasma and serum samples were collected from sacrificed animals to obtain routine blood analysis using an automatic blood analyzer (XN-1000 V, Sysmex Co. Ltd., Japan) and blood biochemical analysis using an automatic biochemical analyzer (HITACHI 7020, HITACHI Co. Ltd., Japan) and an electrolyte analyzer (AC9800, AUDICOM Co. Ltd., China). The 8-hydroxy-2 deoxyguanosine (8-OHdG) content of urine samples of mice in each group was measured using an ELISA kit (Sangon Biotech Co. Ltd., China). The 8-OHdG content of urine samples of site workers was also measured by this method.

After euthanasia, the heart and aorta of mice were removed under a stereo microscope (SZN71, SDPTOP Co. Ltd., China) and soaked in a 10% formalin solution. The whole heart was cut into 5 μm longitudinal sections and then stained with hematoxylin & eosin (H&E) and a Masson dye solution set (Servicebio) according to standard procedures. The aorta was cut at the ascending position and then stained with hematoxylin & eosin (H&E) and Verhoeff-van-Gieson (VVG) (Servicebio) stain to reveal the presence of inflammatory factors and elastic fibers due to pathological conditions. Cell images were acquired using a DM6B upright microscope (Leica Micro Systems Inc., USA) equipped with 20× and 40× objectives. The fibrosis area in treated and control groups (at least 7 sections from three consecutive slides per mouse) was analyzed using Image-Pro Plus 7.0 software (Media Cybernetics, Rockville, MD, USA).

### 2.6 Extraction and Purification of Total Pb in Tissue Samples

Mouse liver, spleen, lung, kidney, intestine, stomach, muscle, bone, and brain samples were collected and then freeze-dried for 48 h. After weighting, tissue samples were ground using zirconia grinding beads in an automatic sample rapid grinding machine (JXFSTPRP-24, Jingxin Co. Ltd., China) at low temperature (4 °C), and then the suspension was transferred to 8 mL of 68% nitric acid for pre-digestion.^10^ This was followed by microwave digestion and ICP lead content detection, as described in Section 2.2.

### 2.7 Bioavailability

The bioaccumulation curve of PM_2.5_-Pb can be obtained by linear fitting the total pollutant exposure dose and Pb accumulation in tissues.^19^ Under different exposure pathways, RBA of Pb compounds in PM_2.5_ at the smelting site can be defined as the ratio of the Pb concentration in a normalized target tissue after PM_2.5_-Pb exposure to that of Pb(Ac)_2_ as follows:^11,20^
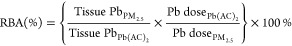
1where RBA( % ) is the bioavailability under specific exposure pathways, Tissue Pb_PM_2.5__ and Tissue Pb_Pb(AC)_2__ are the accumulation concentrations of Pb compounds and lead acetate, respectively, in PM_2.5_ related to the cardiovascular function after exposure, and Pb dose_Pb(AC)_2__ and Pb dose_PM_2.5__ are the corresponding exposure doses of mice, respectively.

### 2.8 Cardiovascular Health Risk Assessment Modeling

The health risks of PM_2.5_-Pb in the smelter workers were investigated using human health risk assessment models specified in the Guidelines for Exposure Assessment.^21^ First, the average daily dose (ADD) was calculated by eq 2:^22^
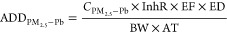
2where ADD_PM_2.5_ – Pb_ is the average daily dose calculation of PM_2.5_-Pb, mg·m^–3^·day^–1^, InhR is the respiratory rate of workers (15.2, m^3^·day^–1^), EF is the daily exposure duration (350 d/a), ED is the annual exposure duration (24 a), BW is the mean body weight (60 kg), and AT is the exposure duration (i.e., 365 × ED).

Since accurate health risk calculation requires a one-to-one correspondence between reference dose (RfD) and the target pollutant PM_2.5_-Pb, we revised the RfD value of PM_2.5_-Pb by the damage threshold calculated from the no observed adverse effect levels (NOAEL) of various toxicological effects. Finally, we revised the health risk assessment formula to include the two parameters RBA and RfD_PM_2.5_ – Pb_ in order to accurately assess the health risk of PM_2.5_-Pb at the smelting site.

### 2.9 Statistical Analysis

Concentrations of PM_2.5_ and PM_2.5_-Pb in the atmosphere were expressed as 24 h average values. Each experiment and detection were repeated at least three times. The results were expressed as mean ± SD. Significant differences between means of different exposure groups were determined using an unpaired student’s *t*-test with GraphPad Prism 8. Pearson’s correlation coefficients were calculated using Origin (version 2022b) and R (ver. 3.6.3) to analyze the correlation of the heavy metal content in samples via different exposure pathways, with *p* < 0.05 set as the threshold for significance.

## 3 Results and Discussion

### 3.1 Contamination Profile and Chemical Speciation of PM_2.5_-Bound Pb in a Smelting Area

**Figure 1:**
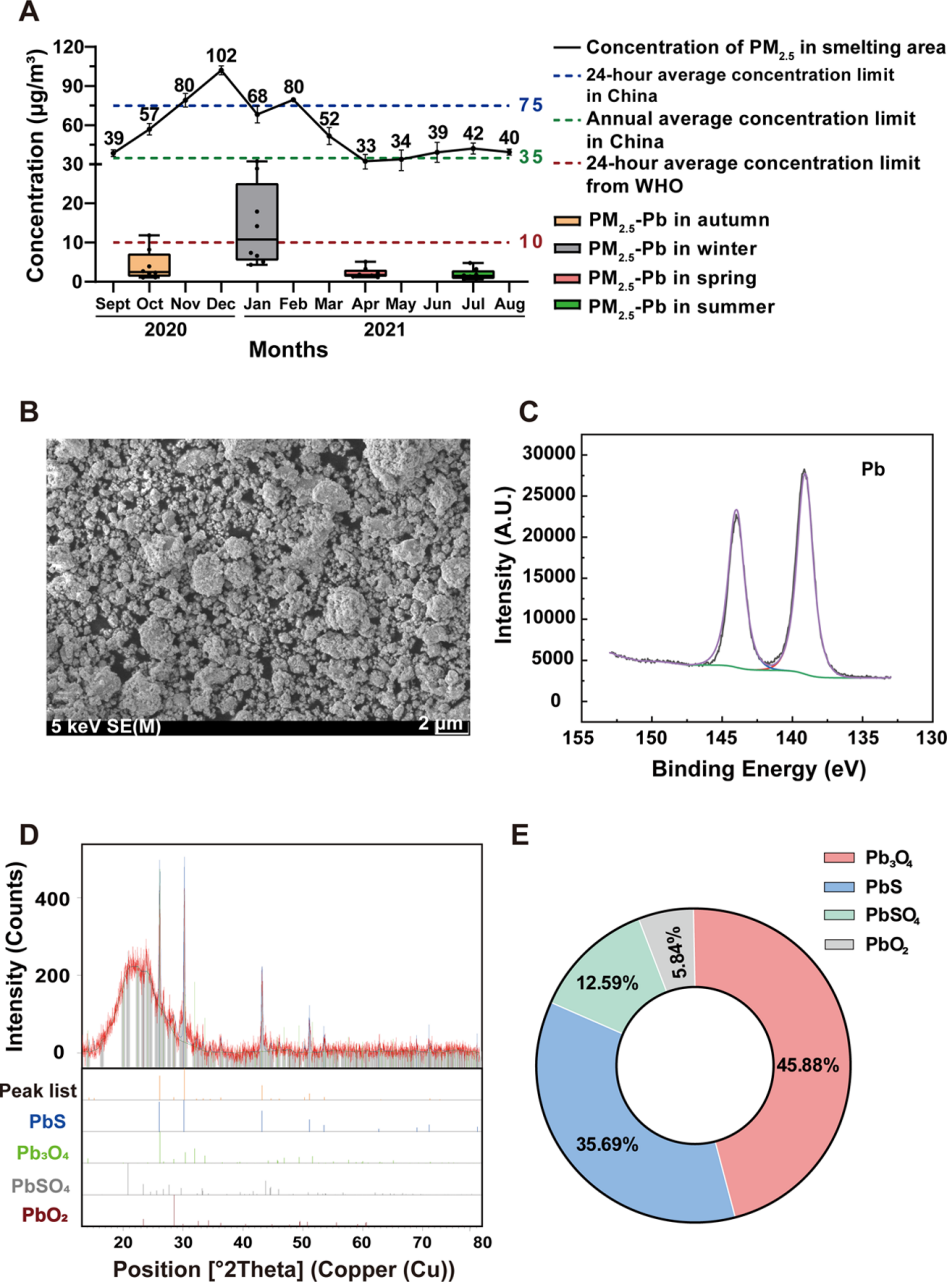
Content and speciation analysis of PM_2.5_-Pb. (A) Concentration of PM_2.5_ and PM_2.5_-Pb in the smelting site in 2020–2021; (B) SEM image of PM_2.5_ particles on the filter membrane; (C) XPS spectra of PM_2.5_-Pb on the filter membrane; (D) XRD patterns of PM_2.5_-Pb on the filter membrane; and (E) occurrence ratio of four compounds obtained from XRD analysis.

The PM_2.5_ concentration was examined during four seasons by calculating the mass difference of the filter membranes before and after sampling. As shown in Figure 1A, the average annual PM_2.5_ mass concentration in the smelting area was 55.4 μg·m^–3^, which exceeded the average annual limit (Air Quality Standards of China, GB3095-2012). The annual average concentration of PM_10_ in the site was 164 μg·m^–3^. Pollution levels were highest in the winter, when the concentrations exceeded the 24 h limit, whereas concentrations were lowest in the summer. The study area is located in a temperate continental climate zone with low precipitation. Emissions related to smelting and climate conditions may have both contributed to the high PM_2.5_ levels and formation. In addition, although the low humidity at the study site did not favor the growth of aerosol particles and PM_2.5_ formation, high levels of dust from the smelting site, roads, surrounding unvegetated land, and tailing dump may have contributed markedly to the high PM_2.5_ atmospheric concentrations compared to similar sites in southern and eastern China.^23,24^

Figure 1A shows the Pb content in PM_2.5_ at the smelting area during a whole year. Comparison of PM_2.5_-Pb concentrations between sites and cities is presented in Supporting Information (Table S4). Like the trend in PM_2.5_ mass concentration across the four seasons, the PM_2.5_-Pb content reached a peak (34.6 μg·m^–3^) in winter. The average annual content of PM_2.5_-Pb was 8.05 μg·m^–3^, about 16 times higher than the limit specified in the standards. A comparison of the heavy metal content in PM_2.5_ in major cities of China is shown in Table S4. The average content of PM_2.5_-Pb measured in factories is 43 times higher than that in cities nationwide.

Figure 1B shows that the particles trapped in the filter membranes were mostly <2.5 μm in diameter, and they had a narrow size distribution. XPS analysis suggested that the atmospheric PM_2.5_–Pb mainly existed in the form of Pb(II) and Pb(IV) (Figure 1C). According to XRD patterns (Figure 1D), the principal mineral components of PM_2.5_-Pb in the atmospheric sampling filter membranes were lead sulfide (PbS), lead tetroxide (Pb_3_O_4_), lead sulfate (PbSO_4_), and lead dioxide (PbO_2_), with specific gravities of 35.7, 45.9, 12.6, and 5.84% (from the analysis of all 26 atmospheric sampling filter membranes) (Figure 1E). Analysis of the XRD patterns and calculation of the compound content are presented in detail in the Supporting Information (Section S6). Moreover, the dominant chemical speciation of PM_2.5_-bound Pb in our study site was consistent with that in the particle matters from other four non-ferrous metal smelting sites located in Sichuan Province (Southwestern China), Hunan Province (Central China), Guangdong Province (Southern China) and Guizhou Province (Southwestern China) (Figure S4). In the control area, the non-ferrous metal related speciation was not detected (Figure S5).

Considering the absence of significant anthropogenic sources of heavy metals within 5 km of the smelter site and the local geographical and climatic conditions, the main pollutant source of Pb at the site was probably bound to PM_2.5_. Subsequent chemical speciation analysis revealed that the chemical composition of PM_2.5_-Pb was highly similar to that of the dust, raw materials, and tailing heaps at the site. Further details are provided in the Supporting Information (Section S7). The high pollution level of Pb in PM_2.5_ is partly due to the rich sources at the smelting site and may be related to the binding capacity of PM_2.5_ to Pb. Several studies have investigated the size distribution characteristics of heavy metals in the particulate matter at lead and zinc smelting affected areas and demonstrated that concentrations of Cd, Cu, Pb, and Zn were dominantly enriched in fine particles.^5,25^

### 3.2 Essential Contribution of PM_2.5_-Bound Pb Exposure to the Human Absorption of Pb

**Figure 2:**
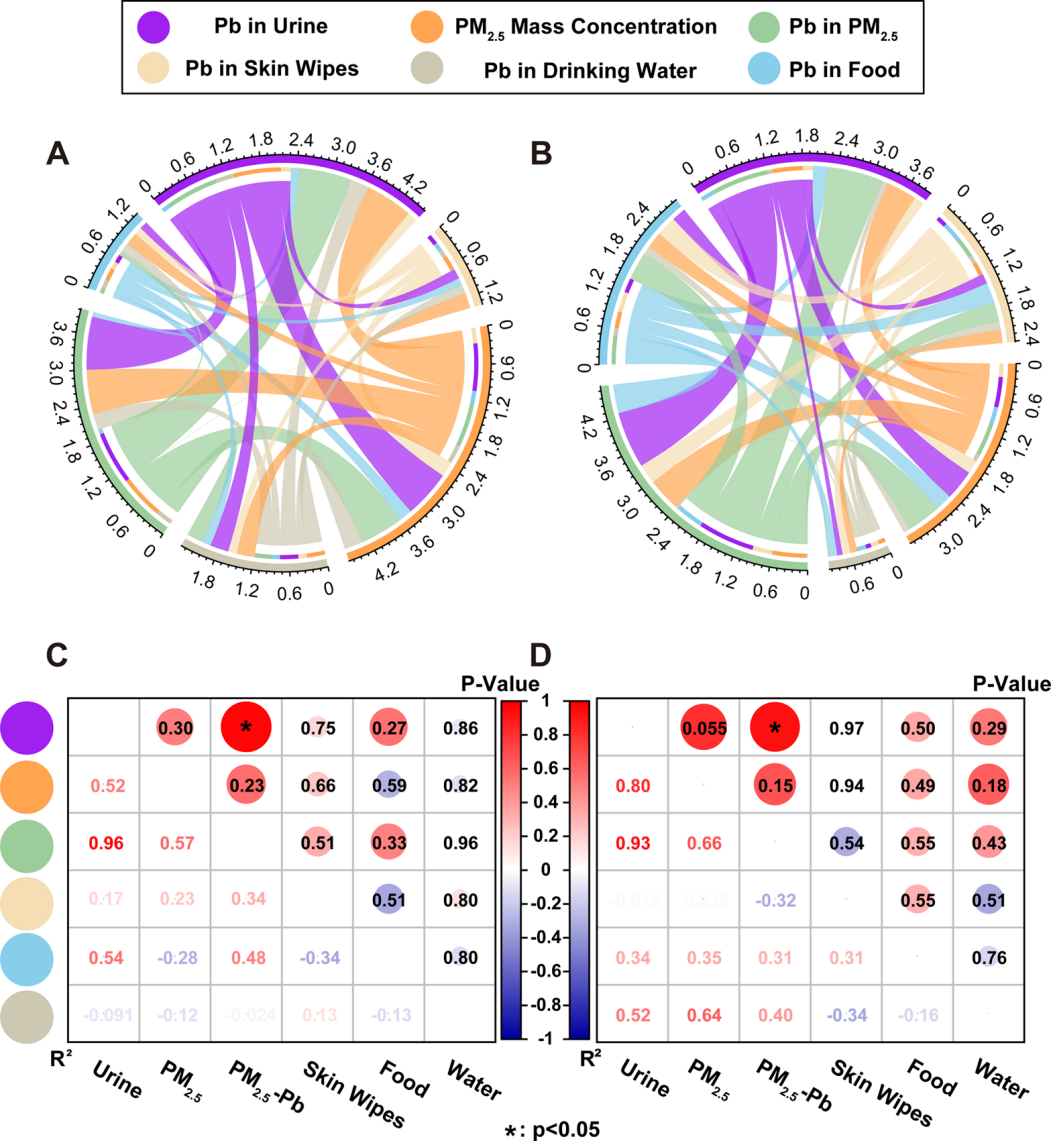
Correlation analysis of the lead content in workers’ urine, PM_2.5_-Pb, skin wipe paper, food, drinking water, and PM_2.5_ mass concentration. (A,C) in summer; (B,D) in winter.

It is vital to identify the dominant medium and pathways for heavy metals to enter the human body in these areas. Therefore, a panel study among smelting area works (Table S7) was performed to directly investigate the contribution of PM_2.5_-Pb exposure to the human absorption of Pb by comparing the association between potential exposure pathways and Pb–U. As shown in Figure 2, in both summer and winter, Pb–U of workers had the highest correlation with the concentration of Pb in PM_2.5_ (*R*^2^ 0.9606 and 0.9276 in summer and winter, respectively), indicating that PM_2.5_-Pb was the main factor contributing to Pb absorption in humans from the environment at the smelting site. Additionally, the seasonal differences in the correlation between Pb–U and PM_2.5_-Pb may be due to the climatic characteristics of the study area that affected the metabolic condition of Pb–U.^26,27^

Compared with the analyzed Pb concentration in whole blood, urine samples are easy to collect and are a non-invasive and economically viable biomonitoring method. Growing evidence has shown that urine concentrations can be an excellent biomarker for the assessment of occupational exposure to Pb in epidemiological studies.^28,29^ In our study, the environment around the site and the participants’ characteristics were relatively stable. Therefore, Pb–U may accurately and conveniently reflect the relationship between workers’ daily intake and environmental exposure.

Previous studies reported that dietary intake was the main pathway of heavy metal exposure for humans in smelting areas.^30,31^ However, our study did not find a strong correlation between Pb in food or water and Pb–U of workers, which may be due to the improvements in China’s food security control and environmental protection measures in the non-ferrous metal smelting industry. Several studies have investigated contamination profiles of various heavy metals in smelting areas and speculated that workers in the non-ferrous industry may be exposed to higher levels of heavy metals mainly through frequent inhalation of contaminated dust or particulate matter.^5,32,33^ Furthermore, studies of trace elements in particulate matter have found that fine particles can carry higher concentrations of toxic metals, such as Cu, Zn, As, Se, Ag, Cd, Ti, and Pb, than coarse particles.^25,34^ In our study, the significant correlation between Pb–U and PM_2.5_-Pb was not only consistent with previous studies but also more directly revealed the important contribution of fine particles at smelting sites to heavy metal exposure of site workers. To the best of our knowledge, this is the first time such an association has been reported for a smelting area. Thus, the findings provide an important scientific basis for identifying targets for pollution detection, safety assessment, and remediation in the modern non-ferrous metal smelting industry.

### 3.3 Bioavailability of PM_2.5_-Bound Pb

**Figure 3:**
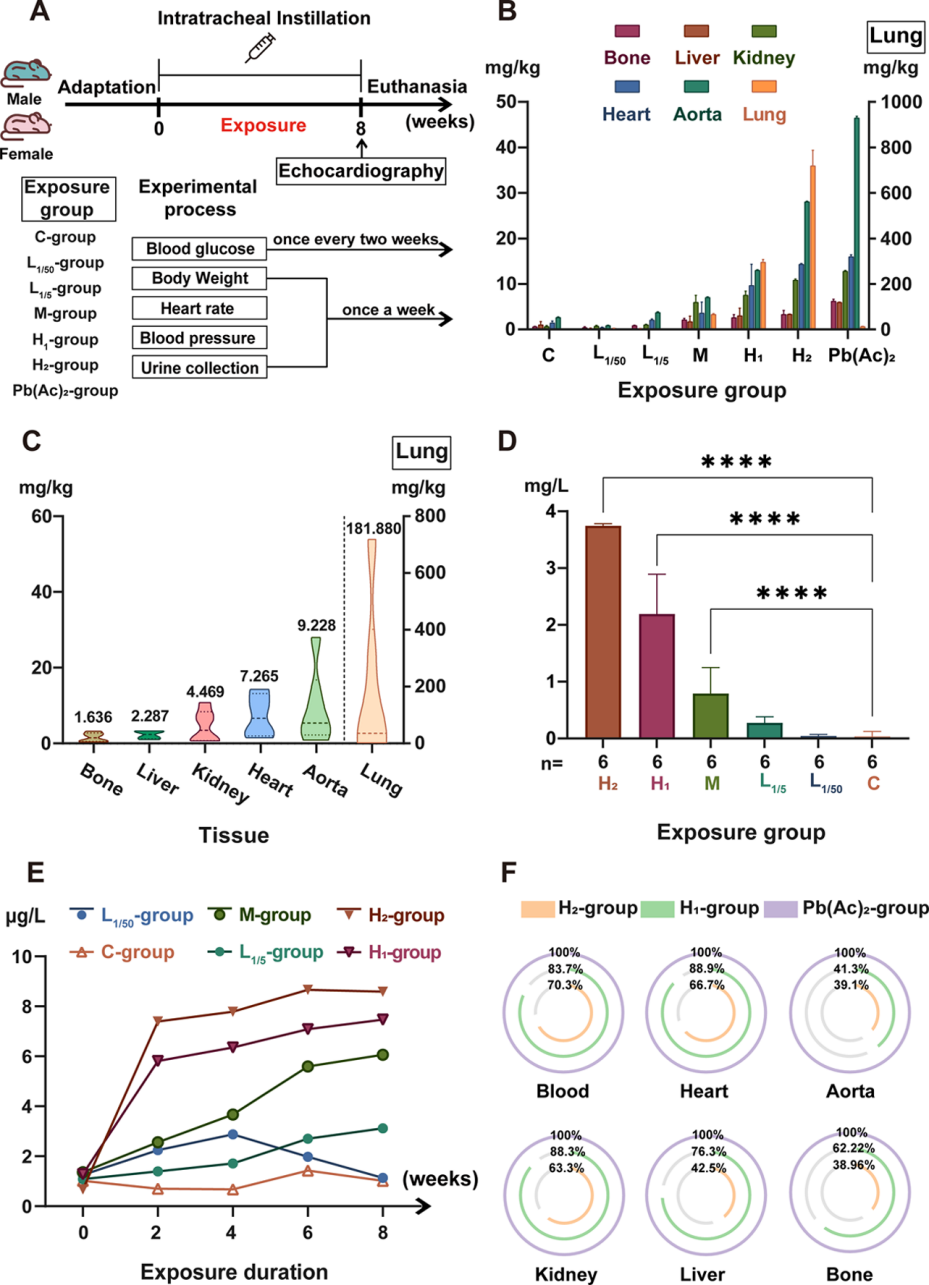
Bioavailability and bioaccumulation analysis of PM_2.5_-Pb. (A) Animal exposure experiment process; (B) lead content in mouse tissues of different exposure groups; (C) lead accumulation in different tissues of mice; (D) blood lead level of mice in different exposure groups; (E) lead content in the urine of mice during the exposure experiment; and (F) bioavailability of PM_2.5_-Pb in different tissues of mice. Values are mean ± SD of six mice. ****p vs C-group <0.0001.

The bioaccumulation and RBA of PM_2.5_-Pb, based on its environmental concentration, composition, and speciation, were estimated using an animal study of mice. The exposure procedure and groups are shown in Figure 3A. After inhalation exposure to a mixture of four chemical species of Pb for 8 weeks, total Pb concentrations in mouse main tissues, blood, and urine were measured (Figure 3B–E). The accumulation levels of Pb in the tissues of the high dosage groups decreased in the order lung > heart > aorta > liver > kidney > bone. There was no significant difference in Pb accumulation between the low dosage groups and the control group, whereas Pb concentrations in the aorta and kidney were relatively high in the medium dosage group (Table S8 and Figure 3B). Additionally, lung tissue had the highest Pb accumulation in all three exposure groups, with average concentrations of 296 μg·g^–1^ dry weight in the H_1_-group (Figure 3C). However, accumulation of Pb in the Pb(Ac)_2_-group was the least (Table S8 and Figure 3B). The accumulation difference between PM_2.5_-Pb and Pb(Ac)_2_ in the lung can be explained by a mathematical model based on particle deposition, where fine particles in the range 3 nm to 1 × 10^4^ nm are primarily deposited by sedimentation in bronchioles and alveoli, but water-soluble chemicals and ultrafine particles smaller than 3 nm particles are hardly deposited by sedimentation in lung airways.^35,36^ To estimate the amount of lead that could accumulate in mouse tissues after exposure to the simulated PM_2.5_-Pb particulates for a long time, we expressed the relationship between the dose of simulated PM_2.5_-Pb and Pb content in tissue using eq 3 based on linear fitting results (Figure S6):
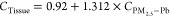
3where *C*_Tissue_ is the accumulation of Pb in mouse tissues and *C*_PM_2.5_ – Pb_ is the total exposure dose of PM_2.5_-Pb.

As shown in Figure 3D, comparison with the C-group revealed that no significant Pb accumulation was observed in the blood of the low dosage groups (L_1/50_- and L_1/5_-groups). However, significant Pb accumulation was observed in the blood of the M- and H-groups, with the concentration depending on the exposure dose. The absorption of PM_2.5_-Pb in mice was continuously observed by detecting the Pb content in urine (Figure 3E). After 6 weeks of exposure, total Pb–U in the H_2_-group reached a steady state, indicating that the amount of Pb absorbed in the body and discharged by metabolism had reached an equilibrium. Similar to the Pb content in blood, Pb–U of mice showed a dependence on exposure dose, confirming the feasibility of using Pb–U as a marker to track human Pb exposure levels. In addition, there were no significant differences in the accumulation of Pb in different tissues and urine between male and female mice except for blood (Figure S7). Therefore, the bioavailability was subsequently calculated without distinguishing gender differences.

The inhalation relative bioavailability of Pb (RBA-Pb) after 8 weeks of exposure was calculated according to eq 1.^11^ Lead acetate was selected as the standard in the equation since the bioavailability of Pb in it is considered to be 100%.^37^ In the H_1_-group, RBA-Pb in the heart, blood, and kidneys was relatively high (88.9, 88.3, and 83.7%, respectively), followed by the liver (76.3%), bone (62.2%), and aorta (41.3%) (Figure 3F). Notably, RBA-Pb in the H_2_-group was the lowest among all groups because of its relatively high exposure concentration and equilibrium state. RBA-Pb in the lungs was not considered in our study due to its extremely high value (Figure 3C). As an inhalation route, lungs are not only sites where pollutants may enter the circulation system but may also be important “storage pools”, where large amounts of insoluble fine particulate matters become trapped and accumulate.^35^ Thus, the large accumulation of PM_2.5_-Pb in the lungs could not be used to calculate its bioavailability. The bioavailability of medium and low dosage group tissues was also not considered owing to its unusually high value. A possible explanation for this might be that the large exposure dose gap between these groups and the Pb(Ac)_2_-group led to differences in human absorption ratios. Interestingly, our results demonstrate that different exposure dosing schemes may yield different bioavailability values over the same exposure period when the target contaminant does not reach absorption equilibrium in animal models. Therefore, the bioavailability of contaminants with long-term exposure effects should be measured at the absorption equilibrium period. Previous studies have indicated that the bioavailability of heavy metals in Pb smelting areas is low, which might be due to the high proportion of sparingly soluble species of the metals in samples from these areas.^38,39^ However, in our study, the inhalation exposure of PM_2.5_-Pb showed a higher biological absorption effect. The average tissue and blood bioavailability reached 60.2% in the H_2_-group, which may be related to the exposure route, dose, chemical structure, and particle size.^18,40,41^ The inhalation RBA-Pb in simulated atmospheric fine particles in Zhong et al. was larger than our value of RBA-Pb in the same tissue (kidney).^11^ This inconsistency may be due to the large differences in exposure time and dose. A higher RBA-Pb value indicates a higher health risk.

### 3.4 Adverse Effects on the Cardiovascular System

According to data from the health questionnaire and physical examination reports, workers in the smelter area had some degree of cardiovascular health risk (Table S9). The proportion of workers with abnormal cardiopulmonary indexes reached about 1/4. Echocardiogram (ECG) examination showed that 19.0% of workers had tachycardia, bradycardia, or arrhythmia and 6.50% of workers had hypertension. Based on the physiological and biochemical indexes and personal lifestyle data obtained from the health questionnaire and physical examination reports, the 10 year ASCVD risk was calculated using China-PAR. Based on these results, 74 (79.8%) participants were classed as low risk and 19 (20.3%) were classed as medium-risk. Further analysis showed that there was a strong correlation between the blood Pb concentration and cardiovascular risk (Table 1). The level of cardiovascular risk at the study site was also significantly higher than the national average.^42,43^ Therefore, based on the analysis of bioavailability, we employed the mouse model to investigate the damaging effects of PM_2.5_-Pb on the cardiovascular system.

**Table 1:** Preliminary Risk Assessment of the Cardiovascular Disease in Site Workers Based on the Prediction for the ASCVD Risk in China (China-PAR)

risk assessment		mean (SD)	*n* (%)	95% CI	*p*-value
10 year ASCAD risk	low risk	1.93 (1.03)	74 (79.8%)	1.37–2.50	<0.05
	moderate risk	7.53 (1.19)	19 (20.3%)	5.34–9.71	
ASCVD risk	low risk	21.5 (5.24)	79 (85.0%)	18.8–24.3	<0.05
	high risk	38.8 (7.28)	14 (15.1%)	16.7–61.0	

**Figure 4:**
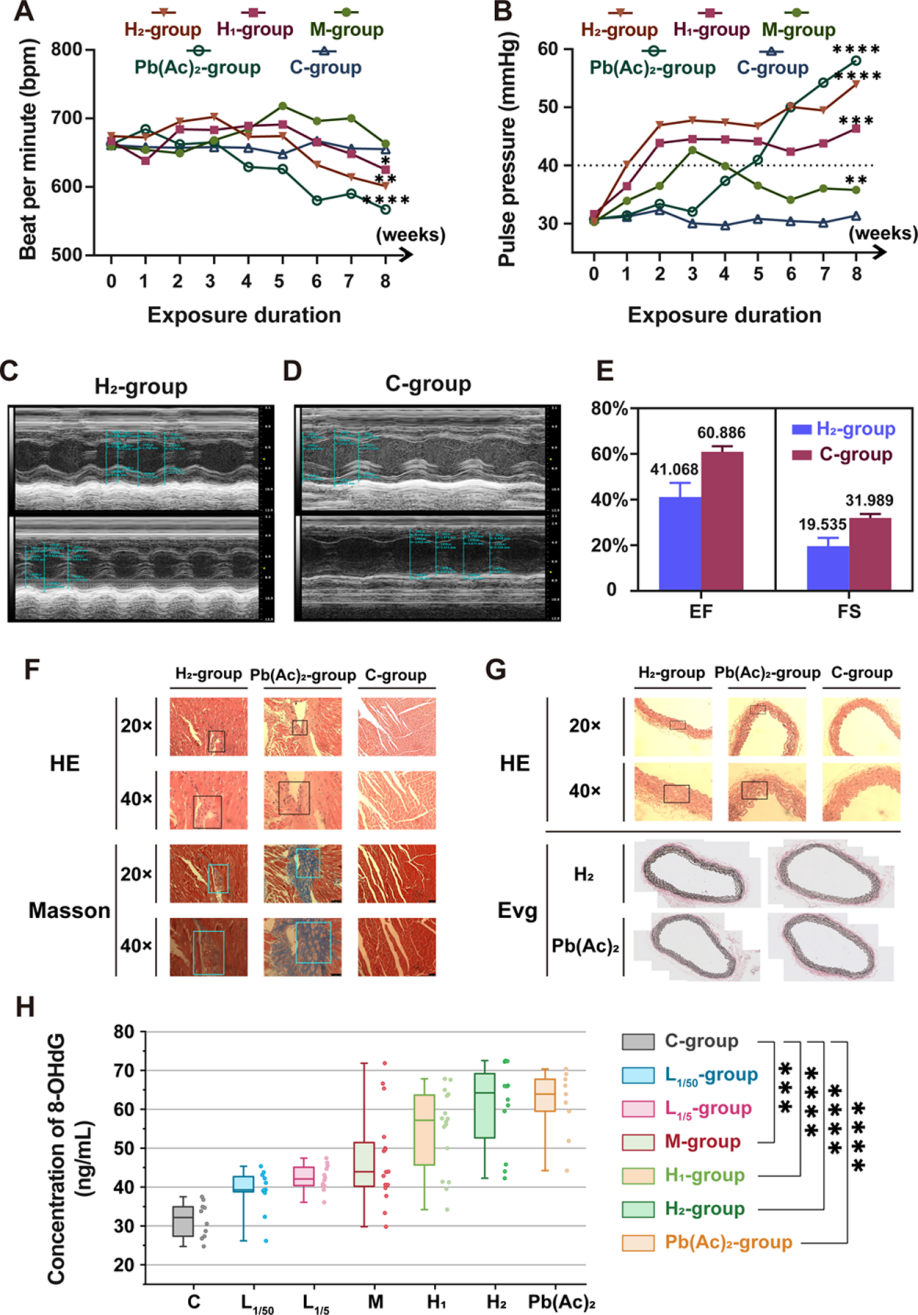
In vivo toxicological effects and histopathology analysis of PM_2.5_-Pb. (A) Heart rate of mice in different exposure groups; (B) pulse pressure of mice in different exposure groups; (C,D) echocardiograms of mouse heart in the H_2_-group and C-group; (E) comparison of EF and FS between the H_2_-group and C-group; (F) representative histopathological images of mouse heart (magnification 20×, 40×); (G) representative histopathological images of mouse aorta (magnification 20×, 40×); and (H) concentration of 8-OHdG in the urine of mice in different exposure groups. Values are mean ± SD of six mice. ***p < 0.001 vs C-group, ****p < 0.0001.

The heart rate and blood pressure of mice were recorded weekly (Figure 4A,B). The heart rate of the high dosage groups and Pb(Ac)_2_-group initially increased but then decreased. The Pb(Ac)_2_-group showed the earliest and most obvious decrease, consistent with our previous studies.^15,44^ The heart rate of the M-group increased from the third week and peaked at the fifth week, before returning to the initial level at the end of the exposure, which may be due to the body’s ability to repair reversible damage and functional recovery.^45,46^ There were no significant differences in heart rate among the low dosage groups. However, both the Pb(Ac)_2_-group and H_2_-group showed significant differences from the C-group in the last week of exposure (*p* < 0.0001 and *p* = 0.0075, respectively), suggesting that PM_2.5_-Pb can damage the cardiovascular system and cause an abnormal heart rate.^47^ Low concentrations of Pb from atmospheric particles have been shown to have a significant effect on blood pressure.^47,48^ Consistent with the heart-rate data, the pulse pressure of mice showed a gradual upward trend, except in the C-group. This indicates that the effect of PM_2.5_-Pb on pulse pressure also depends on exposure concentration.^49^ During the last week of exposure, the pulse pressure of the Pb(Ac)_2_-group (*p* < 0.0001), H_2_-group (*p* < 0.0001), and H_1_-group (*p* = 0.0008) exceeded 40 mmHg and showed a significant difference from the C-group. The H_2_-group and H_1_-group exceeded 40 mmHg in the second week, earlier than the other groups. After the heart rate and blood pressure were found to be abnormal in the H_2_-group, we performed echocardiography on mice with possible cardiovascular damage (Figure 4C,D). The left ventricle ejection fraction (EF) and left ventricle fractional shortening (FS) were used to assess the cardiopulmonary function of mice (Figure 4E).^50,51^ Both EF and FS were lower in the H_2_-group than in the C-group, showing that the heart function of mice in the high dosage groups was more seriously damaged by the higher Pb content in tissue.

Inflammation was also detected in the high dosage groups and Pb(Ac)_2_-group (Figure 4F,G). Since a large number of heavy metals may contaminate the environment in non-ferrous metal areas, the main route of exposure of these heavy metals to the human body and their potential adverse effects was investigated. The significant contribution of PM_2.5_-Pb exposure to human absorption of Pb is likely associated with significant cardiovascular damage induced by PM_2.5_-Pb. Similar studies have reported that heavy metal concentrations in PM_2.5_ correlate with the occurrence of cardiovascular diseases.^47,52^ Urinary 8-OHdG is a major biomarker of oxidative stress.^31^ We detected elevated concentrations of 8-OHdG in the mouse urine of the high dosage groups (Figure 4H), indicating that PM_2.5_-Pb may react with specific enzymes and other biological macromolecules in the body to induce a redox imbalance, leading to cardiovascular oxidative stress and an inflammatory response, which is the main mechanism of cardiovascular damage associated with PM_2.5_-HMs.^3,52^

### 3.5 Cardiovascular-Specific Health Risk Assessment

Bioavailability is an important parameter when considering in vivo toxicity and health risk.^10^ In this study, we established a correlation between the PM_2.5_-Pb concentration in the atmosphere and bioavailability in order to determine the end point of the absorption balance of Pb in vivo. Based on the Pb content in urine (Figure 3E), the H_2_-group was the only group to reach equilibrium. In this group, the absorption and metabolism of PM_2.5_-Pb in mouse tissues were equal, and their bioavailability was relatively stable. Therefore, we determined that the RBA in eq 3 was the relative bioavailability of the H_2_-group, which was 60.2%.

**Figure 5:**
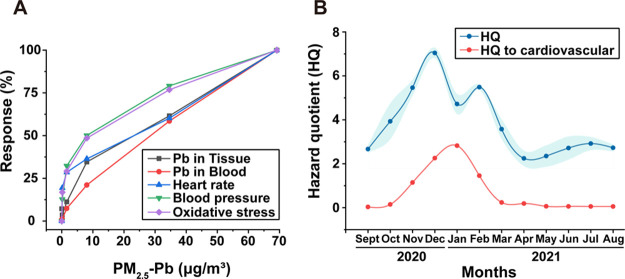
Construction of a cardiovascular-specific health risk assessment model. (A) Response of various cardiovascular damages at different exposure concentrations; (B) comparison of the hazard quotient calculated before and after using the modified model.

After verifying the RBA-modified method for PM_2.5_-Pb, we calculated the corresponding RfD according to the in vivo toxicological exposure experiment results. By analyzing three fitting curves of different toxicological effects (Figure 5A), we found that the M-group had no obvious toxicological effects, whereas the H_1_-group had obvious effects. We determined the NOAEL of PM_2.5_-Pb in the smelting site based on the damage effect and calculated the corresponding RfD (Section S8). The value corresponds to PM_2.5_-Pb concentrations in the environment as a healthy baseline in the atmosphere (3.01 μg·m^–3^). Thus, when the concentration of PM_2.5_-Pb in the atmosphere reaches this value or more, it may cause damage to the human body. Target contaminants and RfD used in traditional health risk assessments are unsuitable for assessing this type of health risk as the results are often too high and unconvincing.^53^ Therefore, we attempted to accurately determine the hazard quotient (HQ) corresponding to PM_2.5_-Pb using the RBA and damage thresholds obtained from the in vivo experiments to ensure a one-to-one correspondence between the target pollutant (PM_2.5_-Pb) and RfD and HQ:
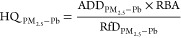
4where RfD_PM_2.5_ – Pb_ is the reference dose of PM_2.5_-Pb (mg·kg^–1^·day^–1^), HQ_PM_2.5_ – Pb_ is the HQ of PM_2.5_-Pb, and RBA is the relative bioavailability of PM_2.5_-Pb (assumed to be the average RBA for the H_2_-group, i.e., 60.2%).^21,54^

Figure 5B compares HQ values before and after using the modified model. Without the modifications, the PM_2.5_ concentration was used as the concentration of the target pollutant, and a high non-carcinogenic risk was predicted for workers in the smelter throughout the year. However, this was not supported by the collected physical examination reports. After implementing the modifications, only November, December, January, and February showed non-carcinogenic risks to workers, associated with the PM_2.5_-Pb content (Figure 1A) and the minimum effective environmental concentration corresponding to the damage threshold calculation (3.01 μg·m^–3^). Thus, our modified model enables accurate non-carcinogenic risk assessment of workers at smelter sites exposed to PM_2.5_-Pb via inhalation.

However, the model cannot be used to identify other exposure routes, e.g., ingestion and dermal contact, which should also be determined to assess PM_2.5_-HM exposure because of the diversity of human exposure pathways and individual differences. Additionally, further studies are needed to assess the health risks via the integration of multiple pollutants carried by PM_2.5_ at smelting sites.

## Supporting Information Available

Specific preprocessing and pre-detection methods of filter membranes, quality assurance and quality control (QA/QC); SEM of the simulated PM_2.5_ suspension; calculation of exposure dose in the animal experiment; echocardiogram of the mice of the H_2_-group and C-group; XRD analysis process; chemical composition of Pb in PM_2.5_, dust, and tailing heaps; calculation process of RfD; ICP-MS detection conditions; simulated PM_2.5_ drip suspension configuration; ejection fraction and fractional shortening measured in the H_2_-group and C-group; comparison of the PM2.5-Pb value between the study site and national city in China; approximate mass ratio of PM_2.5_-Pb compounds measured in sites by season; existence forms and mass proportion of PM_2.5_-Pb according to the XRD results; characteristics of panel study participants; concentrations of lead in various tissues of C57BL/6 mice in five exposure groups; occupational physical examination report, questionnaire analysis based on 93 workers; distribution of sampling points at the non-ferrous metal smelting site; SEM image of simulated PM_2.5_ suspension at a magnification of 1.00 kx and 2.00 kx; main speciation of lead in the dust from the non-ferrous metal smelting sites located in the Gansu Province (Northwestern China), Sichuan Province (Southwestern China), Hunan Province (Central China), Guangdong Province (Southern China), and Guizhou Province (Southwestern China), respectively; main speciation of PM_2.5_-Pb in the non-ferrous metal smelting site and background area; linear fitting of the dose of simulated PM_2.5_-Pb and lead content in tissue; and lead content in blood—male and female (PDF)
