# Supplementary material for: Contribution and Effects of PM2.5-Bound Lead to the Cardiovascular Risk of Workers in a Non-Ferrous Metal Smelting Area Considering Chemical Speciation and Bioavailability
Source: Environ Sci Technol. 2023 Jan 23;57(4):1743–54. doi: 10.1021/acs.est.2c07476 (PMC10775141; doi:10.1021/acs.est.2c07476)
Supplement: Supplementary file 1 — es2c07476_si_001.pdf [file es2c07476_si_001.pdf]

Supporting Information for

Contribution and Effects of $PM_{2.5}$-Bound Lead to Cardiovascular Risk of Workers in a Non-Ferrous Metal Smelting Area Considering Chemical Speciation and Bioavailability

Xiaochong He[a], Qiting Zhao[a], Xuyang Chai[a], Yuanyuan Song[b], Xuelan Li[c], Xingwen Lu[a], Shoupeng Li[d], Xin Chen[c], Yong Yuan[a], Zongwei Cai[a,b*], Zenghua Qi[a*]

[a]Guangdong-Hong Kong-Macao Joint Laboratory for Contaminants Exposure and Health, School of Environmental Science and Engineering, Institute of Environmental Health and Pollution Control, Guangdong University of Technology, Guangzhou, 510006, China
[b]State Key Laboratory of Environmental and Biological Analysis, Department of Chemistry, Hong Kong Baptist University, Hong Kong, China
[c]The Center for Reproductive Medicine, Shunde Hospital, Southern Medical University (The First People's Hospital of Shunde), 528300, Foshan, Guangdong, China.
[d]Analysis and Test Center, Guangdong University of Technology, Guangzhou 510006, China

*Corresponding author:
Zongwei Cai, Ph.D.
Department of Chemistry
Hong Kong Baptist University
Hong Kong, China
Tel: 852-34117070
Fax: 852-34117348
E-mail: zwcai@hkbu.edu.hk

Zenghua Qi, Ph.D.
Rm 510, Engineering Facility Building No.3
School of Environmental Science and Engineering
Guangdong University of Technology
Guangzhou, China
Tel: 020-39322292
Fax: 020-39322292
E-mail: zenghuaqi@gdut.edu.cn

Contents:

Supplementary text

S1. The specific preprocessing and pre-detection methods of filter membranes
S2. Quality assurance and quality control (QA/QC)
S3. SEM of the simulated $PM_{2.5}$ suspension
S4. Calculation of exposure dose in animal experiment
S5. Echocardiogram of the mice of H_2-group and C-group
S6. X-ray diffraction (XRD) analysis process
S7. The chemical composition of Pb in $PM_{2.5}$, dust and tailings heaps
S8. The calculation process of RfD

Table S1 – Table S9

Table S1. ICP-MS detection conditions
Table S2. Simulated $PM_{2.5}$ drip suspension configuration
Table S3: The ejection fraction and fractional shortening measured in H_2-group and C-group
Table S4: Comparison of the $PM_{2.5}$-Pb value between study site and national city in China
Table S5: Approximate mass ratio of $PM_{2.5}$-Pb compounds measured in sites by season
Table S6: Existence forms and mass proportion of $PM_{2.5}$-Pb according to the XRD results
Table S7: Characteristics of panel study participants
Table S8: Concentrations of lead in various tissues of C57BL/6 mice in five exposure groups
Table S9: Occupational physical examination report, questionnaire analysis based on 93 workers

Figure S1 – Figure S7

Figure S1. The distribution of sampling points at the non-ferrous metal smelting site
Figure S2. An SEM image of simulated $PM_{2.5}$ suspension at a magnification of 1.00 kx
Figure S3. An SEM image of simulated $PM_{2.5}$ suspension at a magnification of 2.00 kx
Figure S4. The main speciation of lead in the dust from the non-ferrous metal smelting sites located in the Gansu province (Northwestern China), Sichuan province (Southwestern China), Hunan province (Central China), Guangdong province (Southern China) and Guizhou province (Southwestern China), respectively.
Figure S5. The main speciation of $PM_{2.5}$-Pb in the non-ferrous metal smelting site and background area
Figure S6. Linear fitting of the dose of simulated $PM_{2.5}$-Pb and lead content in tissue
Figure S7. Lead content in blood- male and female

Supplementary Text

S1. The specific preprocessing and pre-detection methods of filter membranes

The gravimetric analysis based on filter collection was used to confirm the mass concentration of $PM_{2.5}$. All filter membranes were weighed using a micro-electronic balance with an accuracy of 0.01 mg before and after sampling. Ultimately, took the average of three parallel data as the final result, then used the following formula to calculate.

$$C = \frac{(W_2 - W_1) \times 10^6}{V_0} \quad (1)$$

where C stands for the mass concentration of $PM_{2.5}$ in the atmosphere ($\mu g/m^3$), W_1 stands for membrane quality before sampling (g), W_2 stands for membrane quality after sampling (g), V_0 is the sampling volume converted by standard conditions (m^3).

In order to determine the content of main anions in $PM_{2.5}$, we further treated the filter membrane. After ultrasonic extraction in water bath at 70°C for 3 hour, eight water-soluble inorganic ions (K^+, Ca^{2+}, Na^+, Mg^{2+}, Cl^-, SO_4^{2-}, NO_3^-, NH_4^+) in the supernatant were determined by ion chromatography like the previous study. The contents of elemental carbon and organic carbon were quantitatively determined by total carbon analyzer[1].

S2. Quality assurance and quality control (QA/QC)

ICP-MS detection followed the principle of multiple repetitions. The internal standard correction quantitative analysis method was used for determination. Each sample (including atmospheric sampling filter membrane, human urine, skin wipe, food, drinking water, mouse tissue, mouse urine and other samples) was guaranteed to have three repetitions and be detected three times. Samples digestion procedures were carried out under the same conditions of filter blank, reagent blank and high recovery rate. All samples are the same rinse method, transfer method, digestion power and digestion time to ensure that three replications of the one sample were obtained after digestion.

The digestion solution of all samples was pumped into the atomization system by a single-tube peristaltic pump and then entered the plasma torch flame. Before each detection, pure water and 2% nitric acid were used to wash the peristaltic pump for 2 min before the next sample injection. According to the operating rules of ICP-MS instrument, the instrument was adjusted to the best working state. The relevant parameters were set and the peak-hopping mode was used for data acquisition. The specific parameters were shown in Table S1. When the instrument was stable, the standard solution, blank solution and sample solution can be determined in sequence.

The recovery was 95.0% and the accuracy was 3%. The relative percentage difference of parallel samples was within 20.0 % to ensure the accuracy of experimental data.

S3. SEM of the simulated $PM_{2.5}$ suspension

Scanning electron microscope (SEM) was used to confirm the particle size of insoluble lead compounds in simulated $PM_{2.5}$ suspension. Figure S2 and Figure S3 show the morphology of the particle suspension obtained at a magnification of 1.00 kx and 2.00 kx, respectively.

S4. Calculation of exposure dose in animal experiment

We mixed standard powder according to the composition ratio of various lead compounds in $PM_{2.5}$-Pb, 0.001g and 0.08g of the mixture were dispersed into 100mL 0.9 % normal saline to obtain the drip suspension of M-group and H_2-group, and then diluted according to the concentration of other groups. The specific configuration concentration is shown in the Table S2.

S5. Echocardiogram of the mice of H_2-group and C-group

Following the 8 weeks exposure period, mice were screened by echocardiography with a VisualSonics Vevo 2100 high-frequency, high-resolution micro-ultrasound

system (with color-Doppler mode). Cardiac function was evaluated by noninvasive ultrasound echocardiography under light isoflurane sedation (0.5–1.00%) to prevent movement and cardio depression[2]. Mice were evaluated to obtain heart dimension and function measurements, including IVS (interventricular septum, systolic and diastolic), LVID (left ventricular internal diameter, systolic and diastolic), LVPW (left ventricular posterior wall, systolic and diastolic). EF and FS of the mice in H_2-group and C-group were calculated by this dimension and measurements. The results are shown in the Table S3.

S6. X-ray diffraction (XRD) analysis process

Speciation of lead compounds in $PM_{2.5}$-Pb requires XRD patterns and the powder diffraction card (PDF-2 2004)[3]. Table S5 and Table S6 show the speciation and mass proportion of lead in $PM_{2.5}$. First of all, according to the XRD analysis, we find out the four materials which have the great match with the three major XRD diffraction peaks, and get their RIR values through the powder diffraction card. Their respective mass ratios are calculated from the RIR value and the strongest peak of each substance.

Our study and most publications have reported that the chemical composition of $PM_{2.5}$-Pb was highly similar to that of the dust at the non-ferrous metal smelting sites. Therefore, we added other four non-ferrous metal smelting sites located in Sichuan province (Southwestern China), Hunan province (Central China), Guangdong province (Southern China) and Guizhou province (Southwestern China), respectively. By analyzing the Pb speciation in these sites, we found that the main speciation of lead in dust from these sites was consistent with that in our study (Figure. S4).

S7. The chemical composition of Pb in $PM_{2.5}$, dust and tailings heaps

By XRD analysis, we can additionally obtain the chemical composition of the dust

and tailings. The chemical composition of Pb in $PM_{2.5}$, dust and tailing were similar which also including lead sulfide (PbS), lead tetroxide (Pb_3O_4), lead sulfate ($PbSO_4$) and lead dioxide (PbO_2). The specific gravity of dust is about 34.4% (PbS), 43.2% (Pb_3O_4), 14.4% ($PbSO_4$) and 7.99% (PbO_2). At the meanwhile, the specific gravity of tailing is about 35.7% (PbS), 47.4% (Pb_3O_4), 15.8% ($PbSO_4$) and 2.19% (PbO_2).

At the meantime, comparison of site $PM_{2.5}$ samples with background area samples is presented in Figure S5.

S8. The calculation process of RfD

We evaluated the cardiovascular damage by measuring heart rate, blood pressure, and urinary 8-OHdG levels in mice, where we take the last week of data to assess the final exposure effect. Through the index difference research of each group and C-group mice in the last week, the difference rate of different indicators (exceed the standard value) and exposure concentration linear fitting, through the fitting curve to find NOAELs[4]. Coincidentally, the NOAELs for the three effects are all between the concentrations in $L_{1/5}$-group and M-group. Through the calculation of the Eq. (2), we obtained the reference doses corresponding to the three damage levels are 4.33×10^{-3}, 4.44×10^{-3} and 4.42×10^{-3} $mg\cdot kg^{-1}\cdot day^{-1}$, respectively. Finally, we identified the minimum value above, 4.33×10^{-3} $mg\cdot kg^{-1}\cdot day^{-1}$, as the NOAEL of $PM_{2.5}$-Pb to human cardiovascular system in smelting sites, which slightly higher than the NOAEL (3.52×10^{-3} $mg\cdot kg^{-1}\cdot day^{-1}$) of lead (Pb)[5].

$$RfD_{PM_{2.5}-Pb} = \frac{NOAEL}{MF \times UF} \quad (2)$$

where $RfD_{PM_{2.5}-Pb}$ is the reference dose of $PM_{2.5}$-Pb ($mg\cdot kg^{-1}\cdot day^{-1}$), $NOAEL$ is

the No Observed Adverse Effect Level (mg·kg^{-1}·day^{-1}), MF is the modifying factor (set to 1), UF is the uncertainty factor (assumed to be 1 because we accounted for a difference between human and experimental animals in the exposure experiment).

Table

Table S1 – Table S9

Table S1. ICP-MS detection conditions

RF Power	RF Matching	Auxiliary flow	Carrier Gas	Omega Bias	Omega Lens	Cell Entrance	Deflect	Cell Exit	Plate Bias
1.50kW	1.80V	1.50L/min	1.10L/min	-105V	10.1V	-45.0V	3.80V	-62.0V	-60.0V

Table S2. Simulated $PM_{2.5}$ drip suspension configuration

	$Pb(Ac)_2$-group	$L_{1/50}$-group	$L_{1/5}$-group	M-group	H_1-group	H_2-group
The exposure dose of simulated $PM_{2.5}$-Pb per mouse (mg/kg)	0.37	0.001	0.01	0.06	0.25	0.50
Concentration of drip suspension (mg/mL)	0.55	0.002	0.02	0.09	0.37	0.75

Table S3. The Ejection Fraction and Fractional Shortening measured in H_2-group and C-group

Group	Ejection Fraction (EF)			Fractional Shortening		
	value	Mean	SD	value	Mean	SD
H_2-group	39.3	41.1	6.25	18.7	19.5	3.67
	49.5			24.4		
	34.4			15.5		
C-group	63.6	60.9	2.46	33.9	32.0	1.71
	61.4			32.3		
	57.6			29.7		

Table S4. Comparison of the $PM_{2.5}$-Pb value between study site and national city in China

Study area	Content of $PM_{2.5}$-Pb（ng/m³）
Northern China	171.850
Southern China	155.260
Eastern China	251.725
Central China	237.755
Southwestern China	97.7850
Northeastern China	68.1300
Northwestern China	324.670
National average	186.739
Average content measured in smelt site	8044.76

Table S5. Approximate Mass Ratio of $PM_{2.5}$-Pb Compounds Measured in Sites by Season

Season	Composition	RIR (K)	Intensity of the detection peak I (Cts)	C (I/K)	∑C	Mass proportion (C/∑C×100%)
Spring	PbS	15.1	642	42.7	93.8	45.5%
	Pb_3O_4	13.2	538	40.7		43.4%
	$PbSO_4$	7.99	42.0	5.26		5.61%
	PbSO4	16.9	87.0	5.16		5.50%
Summer	PbS	15.1	612	40.7	106	38.3%
	Pb_3O_4	13.2	607	45.9		43.2%
	$PbSO_4$	7.99	114	14.3		13.4%
	PbO_2	16.9	90.0	5.34		5.03%
Autumn	PbS	15.1	149	9.90	29.9	33.1%
	Pb_3O_4	13.2	199	15.1		50.3%
	$PbSO_4$	7.99	17.0	2.13		7.11%
	PbO_2	16.9	48.0	2.85		9.51%
Winter	PbS	15.1	119	7.91	30.5	25.9%
	Pb_3O_4	13.2	188	14.2		46.6%
	$PbSO_4$	7.99	59.0	7.38		24.2%
	PbO_2	169	17.0	1.01		3.30%

Table S6. Existence forms and mass proportion of $PM_{2.5}$-Pb according to the XRD results

Ref. Code	Phase	Composition	RIR (K)	Mass proportion
01-78-1056	Lead Sulfide	PbS	15.1	35.7%
01-85-0859	Lead Tetroxide	Pb_3O_4	13.2	45.9%
01-83-1720	Lead Sulfate	$PbSO_4$	7.99	12.6%
01-72-2440	Lead Dioxide	PbO_2	16.9	5.84%

Table S7. Characteristics of Panel Study Participants

Participants characteristics	Value
Age, Mean ± SD [range] (year)	43.6 ± 6.90 [25.0-57.0]
Male, no. (%)	16.0 (66.7%)
BMI, Mean ± SD [range]	23.4 ± 2.80 [19.9-30.1]
Heart rate, Mean ± SD [range]	78.0 ± 10.0 [66.0-106]
Systolic pressure, Mean ± SD [range]	119 ± 16.0 [85.0-154]
Diastolic pressure, Mean ± SD [range]	81.0 ± 9.00 [68.0-110]

Table S8. Concentrations (μg·g-1, Dry Weight, dw) of Lead in Various Tissues of C57BL/6 Mice in Five Exposure Groups (Mean ± SD)

Concentration	Tissue					
	Bone	Liver	Kidney	Arota	Heart	Lung
M-group	2.10±0.36	1.74±1.17	5.98±1.54	7.07±0.11	3.60±2.41	66.4±3.23
H_1-group	2.61±0.65	3.03±1.65	7.57±0.86	13.0±0.11	9.69±4.64	296±10.9
H_2-group	3.29±0.90	3.35±0.06	10.8±0.23	28.0±0.29	14.3±0.17	719±68.2
$Pb(Ac)_2$-group	6.20±0.44	5.96±0.07	12.8±0.16	46.5±0.38	16.0±0.41	13.0±0.78
C-group	0.58±0.10	1.03±0.55	0.66±0.20	2.65±0.04	1.46±0.40	2.33±0.10

Table S9. Occupational physical examination report, questionnaire analysis based on 93 workers

Occupational physical examination report	Statistics		Questionnaire on Basic Situation of Workers	Statistics	
**Age (years)**			**Smoking status**		
Mean (SD)	43.6	7.47	Never, n (%)	70.0	75.3%
**Sex**			Ever, n (%)	23.0	24.7%
Male, n (%)	54.0	58.1%			
Female, n (%)	39.0	41.9%	**Drinking condition**		
**Body mass index**			Never, n (%)	68.0	73.1%
Mean (SD)	23.8	2.89	Ever, n (%)	25.0	26.9%
**Heart rate**					
Mean (SD)	77.0	9.18	**Exercise and sleep**		
**Blood pressure**			Abnormal, n (%)	32.0	34.4%
Systolic pressure (SD)	120	15.2	normal, n (%)	61.0	65.6%
Diastolic pressure (SD)	80.0	9.77			
**Blood routine**			**Medical history**		
Abnormal, n (%)	60.0	64.5%	Abnormal, n (%)	37.0	39.8%
normal, n (%)	33.0	35.5%	Abnormal in Cardiovascular, n (%)	23.0	24.7%
**Urine routine**			normal, n (%)	56.0	60.2%
Abnormal, n (%)	20.0	21.5%			
normal, n (%)	73.0	78.5%	**Family history of chronic diseases**		
**Blood lead levels**			Abnormal, n (%)	51.0	54.8%
Exceeds standard, n (%)	24.0.	25.8%	Abnormal in Cardiovascular, n (%)	33.0	35.5%
normal, n (%)	69.0	74.2%	normal, n (%)	42.0	45.2%

Figure

Figure S1 – Figure S7

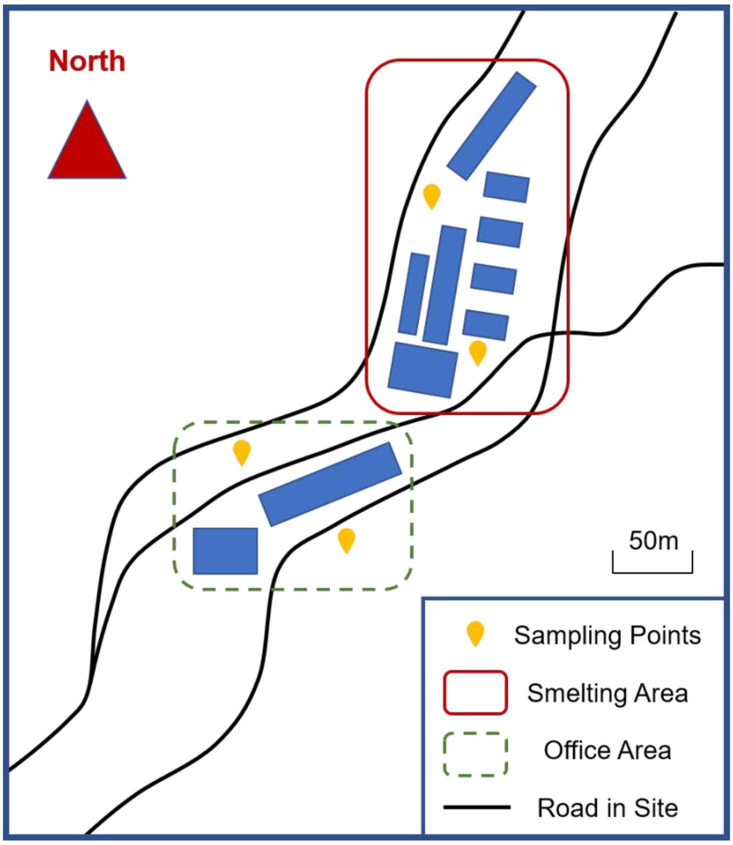

Figure S1. The distribution of sampling points at the non-ferrous metal smelting site.

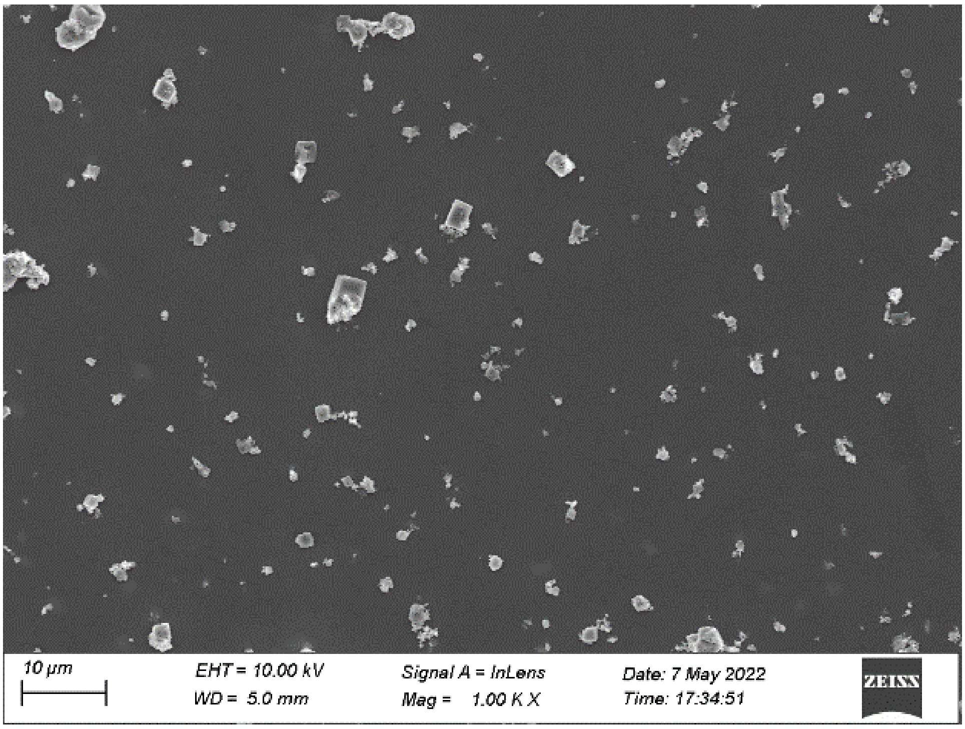

Figure S2. An SEM image of simulated $PM_{2.5}$ suspension at a magnification of 1.00 kx.

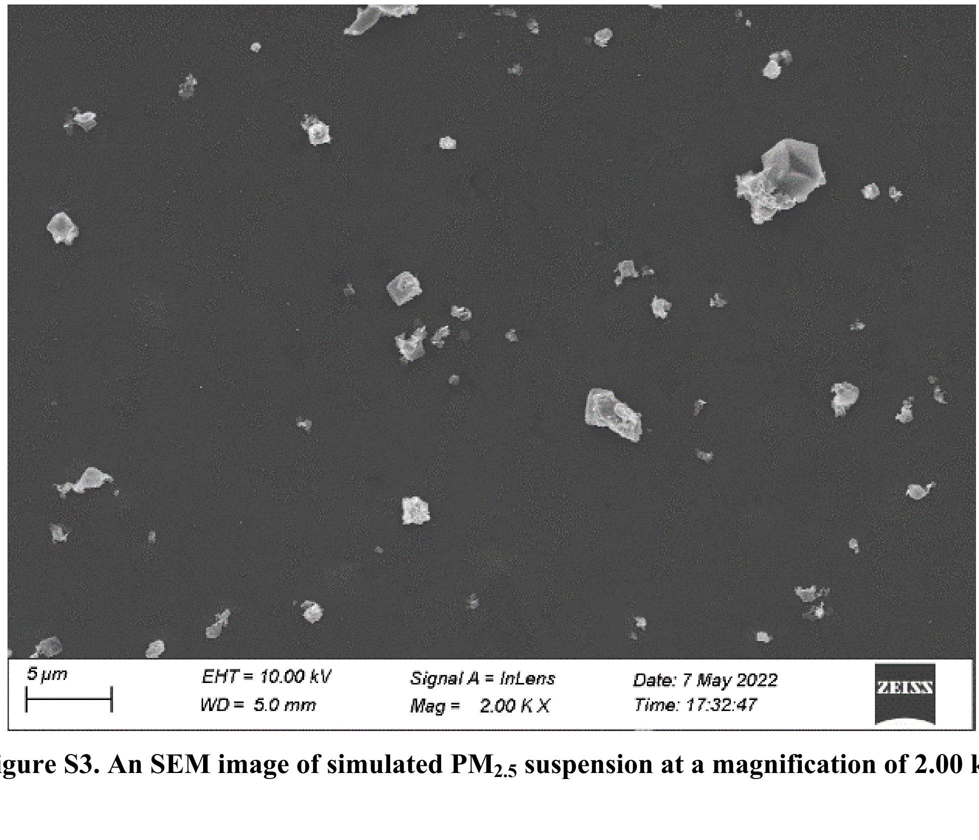

Figure S3. An SEM image of simulated $PM_{2.5}$ suspension at a magnification of 2.00 kx.

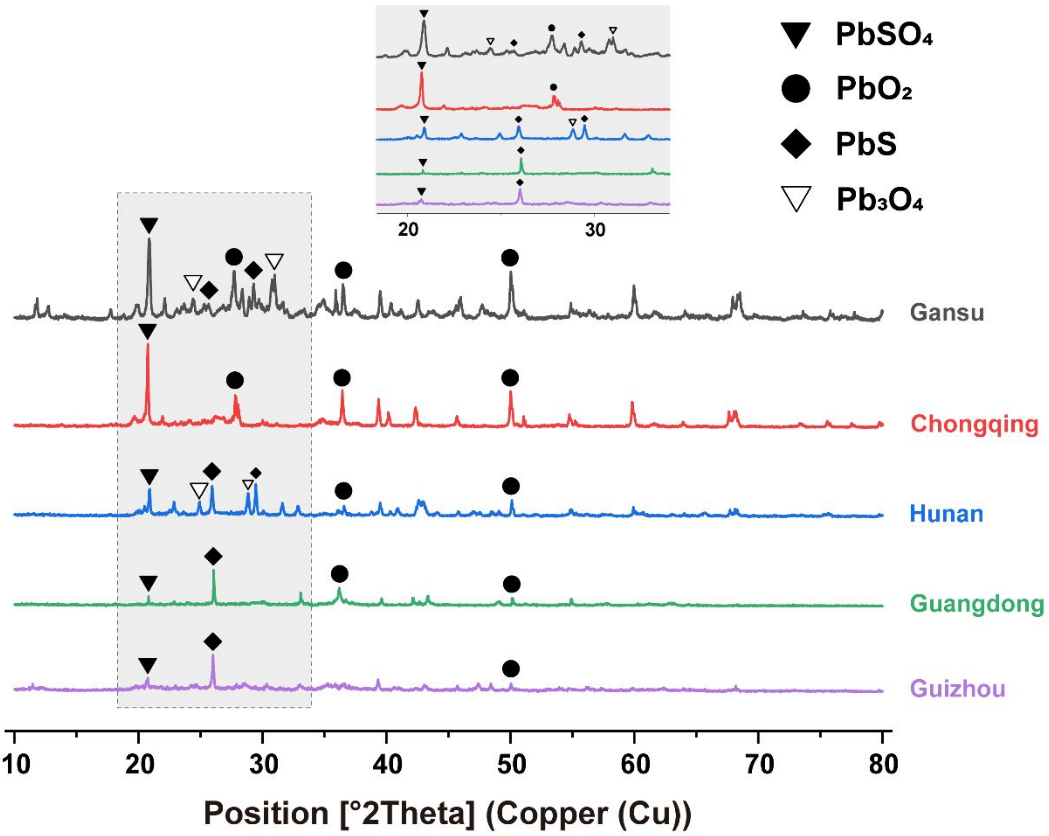

Figure S4. The main speciation of lead in the dust from the non-ferrous metal smelting sites located in the Gansu province (Northwestern China), Sichuan province (Southwestern China), Hunan province (Central China), Guangdong province (Southern China) and Guizhou province (Southwestern China), respectively.

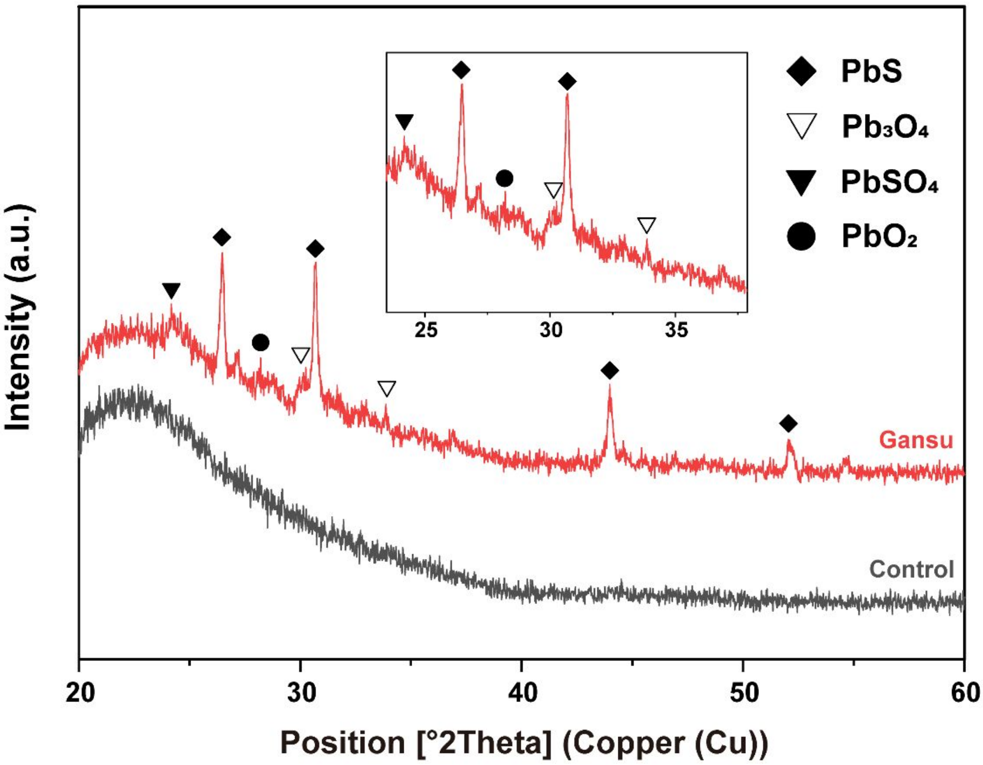

Figure S5. The main speciation of $PM_{2.5}$-Pb in the non-ferrous metal smelting site and background area.

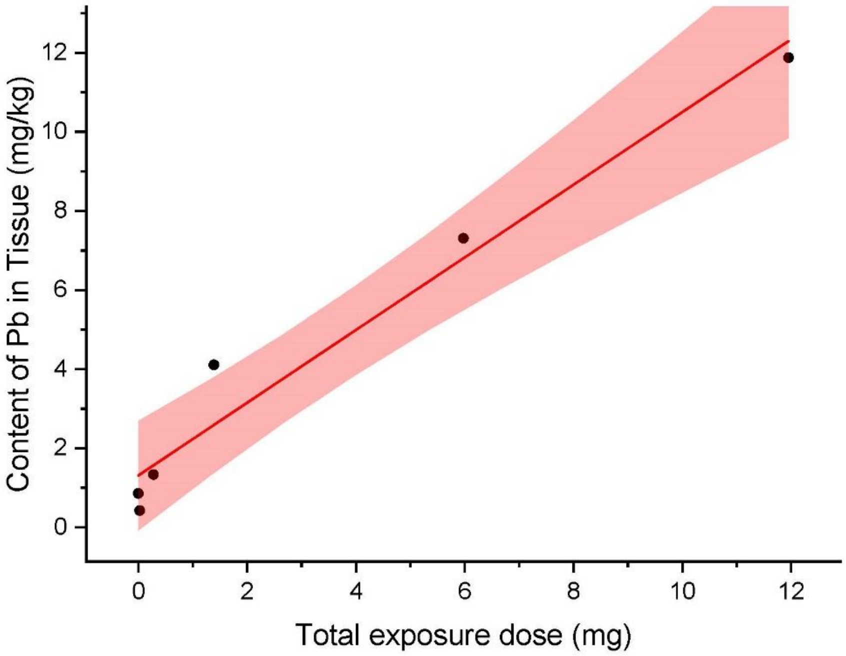

Figure S6. Linear fitting of the dose of simulated $PM_{2.5}$-Pb and lead content in tissue.

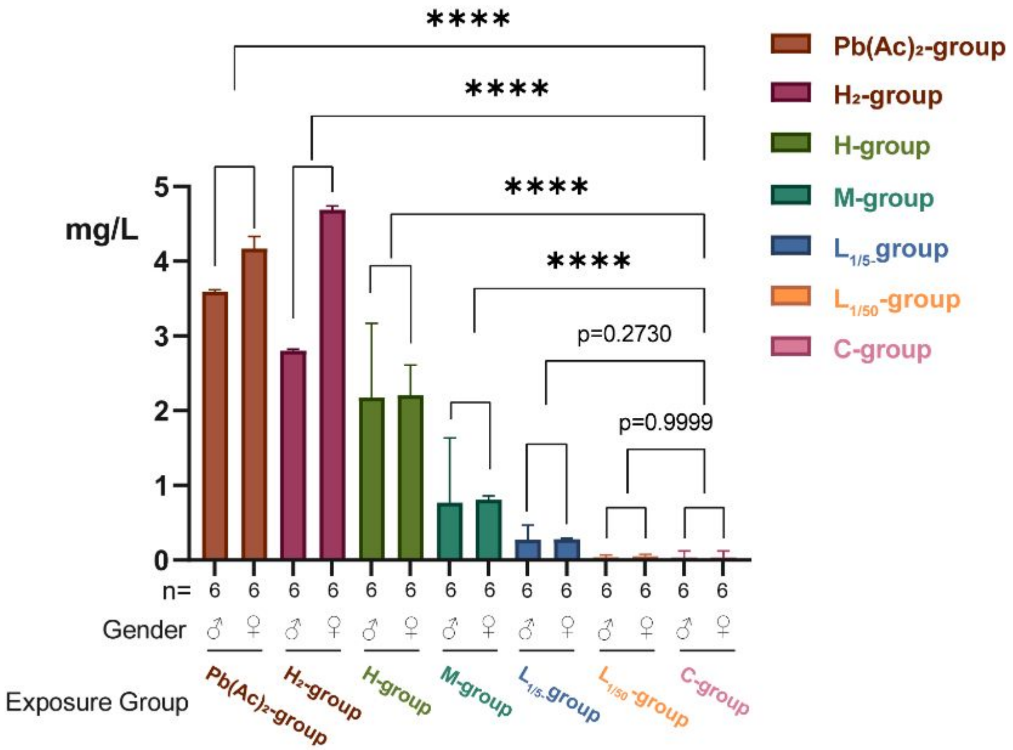

Figure S7. Lead content in blood- male and female.

Reference

1. Wen, L.; Yang, C.; Liao, X.; Zhang, Y.; Chai, X.; Gao, W.; Guo, S.; Bi, Y.; Tsang, S.-Y.; Chen, Z.-F.; Qi, Z.; Cai, Z., Investigation of PM2.5 pollution during COVID-19 pandemic in Guangzhou, China. *J. Environ. Sci.* **2022,** *115*, 443-452.
2. Olfert, I. M.; DeVallance, E.; Hoskinson, H.; Branyan, K. W.; Clayton, S.; Pitzer, C. R.; Sullivan, D. P.; Breit, M. J.; Wu, Z.; Klinkhachorn, P.; Mandler, W. K.; Erdreich, B. H.; Ducatman, B. S.; Bryner, R. W.; Dasgupta, P.; Chantler, P. D., Chronic exposure to electronic cigarettes results in impaired cardiovascular function in mice. *J. Appl. Physiol.* **2018,** *124*, (3), 573-582.
3. ICDD, International Centre for Diffraction Data: Newton Square. *PA* **2002**.
4. USEPA. Methods for Derivation of Inhalation Reference Concentrations and Application Dosimetry. https://www.epa.gov/risk/methods-derivation-inhalation-reference-concentrations-and-application-inhalation-dosimetry (accessed 2021-09-15).
5. USEPA. Risk Assessment Guidance for Superfund: Volume 1 -- Human Health Evaluation Manual (Part A). https://nepis.epa.gov/Exe/ZyPURL.cgi?Dockey=9100UGV0.txt (accessed 2021-09-15).
